# Forty-five-months follow-up of a minimally invasive, interdisciplinary treated hemangioma of the mandible with a high risk of severe bleeding – a case report

**DOI:** 10.1186/s13005-022-00346-x

**Published:** 2023-01-14

**Authors:** Michelle Alicia Ommerborn, Gordon John, Jürgen Becker, Julia Preetz, Sondos Gabris

**Affiliations:** 1 Department of Operative Dentistry, Periodontology and Endodontology, Faculty of Medicine, Heinrich-Heine-University, Düsseldorf, Germany; 2 Department of Oral Surgery and Central Admittance, Faculty of Medicine, Heinrich-Heine-University, Düsseldorf, Germany; 3 Department of Pathology, Faculty of Medicine, Heinrich-Heine-University and University Hospital, Düsseldorf, Germany

**Keywords:** Hemangioma, Mandible, Periapical diseases, Vascular malformation, Case report

## Abstract

**Background:**

Hemangiomas are benign tumours, mostly seen in the soft tissues. The intraosseous appearance is rare, in particular in the jaws they represent a very seldom malformation.

**Aim:**

To present a combined endodontic and surgical management report of a clinical case with a rare intraosseous hemangioma diagnosis in the mandible.

**Case presentation:**

This well-documented case report describes the interdisciplinary treatment approach of an intraosseous hemangioma in the left mandible of a 70-year-old male patient. This incidental finding was detected through a routine dental examination. The panoramic radiograph revealed an asymptomatic, apical translucency approximately 15 mm diameter with contact to the mesial root of the tooth 36. The clinical examinations showed no abnormalities. The multifaceted specialized treatments started with the endodontic treatment of the tooth prior to the surgical removal of the lesion and were followed by the histological assessment. As derived from the histologically verified diagnosis, this rare case included the risk of severe bleeding complications during therapy.

## Background

Vascular anomalies are differentiated into two groups: hemangiomas and vascular malformations. Hemangiomas are not congenital, women are affected twice as often as men, and they show endothelial hyperplasia [1]. The work of Mulliken and Glowacki was accepted by the International Society for the Study of Vascular Anomalies in 1996 and led to the actual accredited classification [2]. Hemangiomas are benign tumours, mostly seen in the soft tissues. The intraosseous appearance is rare, representing 0.5 up to 1 % of all intraosseous tumours [3, 4]. The most frequent locations of these intrabony lesions are vertebra, phalanges and skull [5–8]. Presence in the jaws is uncommon with a predominance of the mandible of 2/3 [9]. More than 50 % of hemangiomas are mainly noticed in the second decade. Women are more frequently affected by these lesions than men with a rate of 2:1 [10–12]. The present report of a very rare case presents a male patient on the 7th decade of life with an intraosseous hemangioma in the left mandible that has been managed in a well-documented minimally invasive interdisciplinary approach.

## Case presentation

**Fig. 1:**
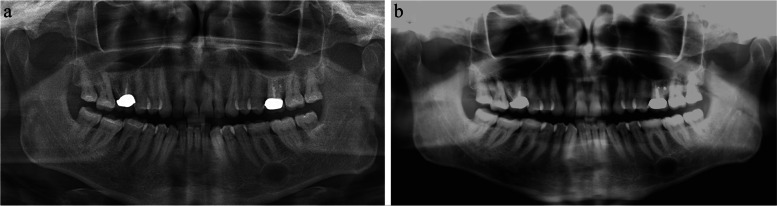
Panoramic radiographs demonstrating the intrabony lesion. **a** Panoramic radiograph 2017. **b** Panoramic radiograph 2011

A 70-year old Caucasian male patient presented for the first time at the department of Operative Dentistry, Periodontology and Endodontology for routine dental control examination in November 2017. He was pain-free, had no history of trauma in the craniofacial area and was otherwise in a good to very good state of health, American Society of Anesthesiologists Physical Status Classification (ASA) [13]. Within the examination, radiographic bitewing images of the left and right side as well as a panoramic radiograph (Sirona Orthophos SL 3D, Dentsply Sirona Deutschland, Bensheim, Germany) were made (Fig. 1a). The image was processed using the software Sirona SIDEXIS 4 (Dentsply Sirona, Charlotte, NC, USA). Apart of the alio loco endodontically treated tooth 16 and additionally root tip resected tooth 26, the x-rays revealed a circular translucency with a cross-sectional dimension of approximately 15 to 20 mm apical to tooth 36. The translucency appeared homogenous, solid, unilocular, and with contact to the mesial root of the tooth 36. Extra-oral examination was unremarkable.

During the following conversation on the radiographic finding, the patient reported that his previous long-time family dentist had already addressed this finding ten years ago. Since he assumed this osteolytic process to be an aneurysmatic bony cyst, he informed the patient that there was no treatment need. In order to receive details regarding the progression and growth of the- at that point unknown- osteolytic process, the patient was asked to provide old panoramic radiographs from the family dentist. At that appointment, sensibility of the mental nerve was tested via discrimination between sharp and blunt and two-point discrimination. The patient was able to identify the applied stimuli correctly in 100 %. The pulp sensibility of the teeth 34, 35, 36, 37 and 38 was also tested and recorded positive for the teeth tested. Indeed, at the second appointment, a panoramic radiograph made in 2011 was submitted by the patient. Here, in general, the osteolytic process looked smaller and, interestingly, the mesial root of tooth 36 did not seem to be in contact to it (Fig. 1b).

**Fig. 2:**
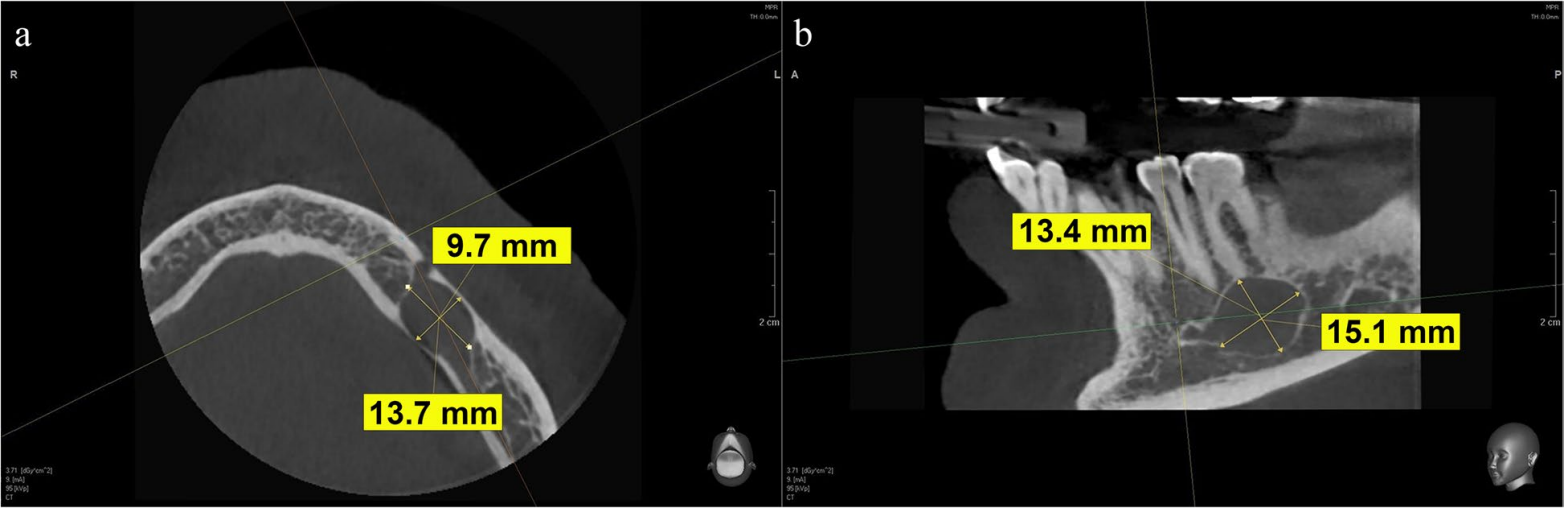
A 5 × 8 cm CBCT of the left mandible. **a** Axial view. **b** Lateral view

In addition to the two-dimensional x-rays, a 5 x 8 cm cone beam computed tomography (CBCT, PaX-i3D, Orangedental & Co.KG, Biberach, Germany) of the left mandible was made providing important details on the topographic relationship of the radiographic lesion and other anatomic structures, such as the inferior alveolar nerve (Fig. 2a, b). In the region of interest, the CBCT showed a homogeneous, unilocular, translucency with a thin sclerotic margin that was not interrupted. The size of the intraosseous lesion was 16 x 12.5 x 10 mm. The continuity of the mandible seemed not to be perforated. The inferior alveolar nerve could not be detected within the lesion. The cranial margin of the lesion was directly in contact to the mesial root of tooth 36, while the distal root seemed to have a bony covering of nearly 0.5 mm. The mental foramen was located directly anterior to the lesions boundary. At this particular time, the most probable diagnosis was that of an infected radicular cyst proceeding from the mesial root of tooth 36. Differential diagnoses such as solitary bone cyst, cystic ameloblastoma or hemangioma had to be discussed as well.

Based on the current CBCT, a decision was made to take a precautionary surgical tissue sample in order to obtain a distinct histopathologically verified diagnosis. Because of the close positional relationship of the intrabony lesion and the roots of tooth 36 with the resulting high risk of devitalization of this tooth, an endodontic treatment was performed before the surgical procedure. Pre-surgical endodontic treatment offers the possibility to remove potentially extruded sealer or obturation material that could potentially irritate the inferior alveolar nerve, during following surgery. Thereby, a second surgery could be avoided. The patient accepted the recommended therapy and oral and written informed consent was obtained for the endodontic-surgical-intervention.

## Endodontic treatment

**Fig. 3:**
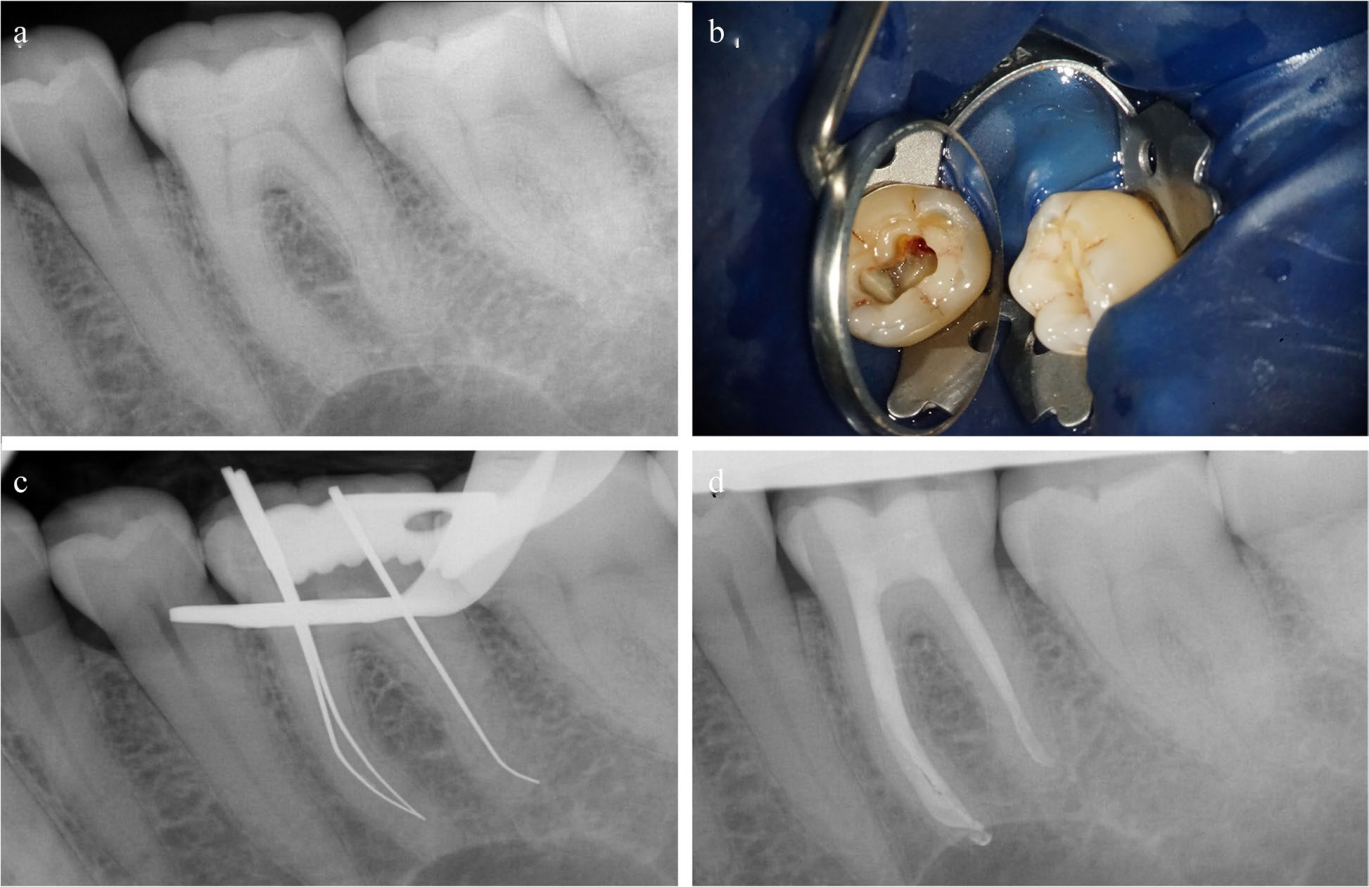
Intra-oral color photograph and periapical radiographs. **a** IOPAR pre-endodontic treatment. **b** Access cavity and depiction of tertiary dentine. **c** IOPAR to control the working length. **d** IOPAR post-endodontic treatment

A repeated pulp sensibility test with CO_2_ of the teeth in the third quadrant revealed a reliable positive sensibility of teeth 34, 35, 36, 37 and 38. Although the CO_2_ sensibility test, in particular, of tooth 36 was positive, the intra-oral periapical radiographic examination (IOPAR, Heliodent DS and Sensor XIOS XG Supreme 0, Dentsply Sirona Deutschland, Bensheim, Germany) revealed a topographical relationship of the mesial root of this tooth to the intrabony lesion (Fig. 3a). The coronal restoration was sufficient for the endodontic treatment to be carried out without replacement. The tooth was anesthetized via infiltration anaesthesia with articaine with 1:200,000 epinephrine as supplement (Ultracain D-S, Sanofi-Aventis, Frankfurt, Germany). The root canal treatment was performed under rubber dam isolation to prevent bacterial re-entry and by using an operating microscope (OPMI PROergo, Carl Zeiss Microscopy, Jena, Germany). During the preparation of the access cavity, tertiary dentin was detected in the area of the mesial pulp horns. With further preparation, three root canals were detected. Interestingly, bleeding was observed in the distal root canal, but not in the mesiolingual and mesiobuccal root canals (Fig. 3b). Mesial and distal canals were initially located with #6, #8 and #10 K-files (Dentsply Maillefer, Tulsa, USA). The canals were irrigated with 3 % sodium hypochlorite (NaOCl) and the working length was determined using the Root ZX apex locator (J. Morita, Tokyo, Japan), radiographic working length verification was obtained with silver points (Fig. 3c). Biomechanical preparation was accomplished with the Mtwo nickel-titanium rotary System (VDW, Munich, Germany). Files were activated using 6:1 reduction VDW handpiece that was powered by a torque-controlled electric motor VDW Gold (VDW, Munich, Germany) The mesiobuccal and mesiolingual root canals were prepared to apical masterfiles ISO 40.04/22 mm and the distal root canal to an apical Masterfile ISO 40.04/18,5 mm. During the root canal treatment, each canal was alternately irrigated with 3 % NaOCl and 17 % ethylenediaminetetraacetic acid (EDTA), followed by re-irrigation with the final rinse 3 % NaOCl using open-ended (29-gauge) irrigation needles (NaviTip, Ultradent, Cologne, Germany). In order to avoid interaction and inactivation of the rinses that may result from their combination, 96 % alcohol was applied as an intermediate rinse. Each rinse NaOCl and EDTA was activated with sonic oscillation tip (EDDY, VDW, Munich, Germany) using SONICflex handpiece (KaVo Dental, Biberach, Germany). After drying the canals, calcium hydroxide (UltraCal XS, Ultadent, Cologne, Germany) was applied as an intracanal medication. The second appointment was five days later, the patient had no complaints and no clinical symptoms. In order to maximize the disinfection effect, each canal received a sonically-activated alternate irrigation with 3 % NaOCl and 17 % EDTA, including an intermediate rinse with 96% alcohol and 3 % NaOCl as a final rinse before root canal filling. Root canal filling was performed with the appropriate Mtwo gutta-percha cones (VDW, Munich, Germany) and AHPlus sealer (Dentsply DeTray, Constance, Germany) using BeeFill 2in1 (VDW, Munich, Germany). After application of a self-etch adhesive system in combination with a silane (Clearfil^TM^ SE Bond, Clearfil^TM^ Porcelain Bond Activator, Kuraray Noritake Dental, Tokyo, Japan), the root canal orifices were sealed with opaque white composite resin (Venus Flow Baseliner, Heraeus Kulzer, Hanau, Germany) and the remaining access cavity was filled with Filtek Supreme XTE composite resin (3M, Seefeld, Germany). IAPOR after the root canal filling revealed a well-tapered and sufficient root canal filling with a minimal extrusion of sealer in the mesial periapical tissues (Fig. 3d).

## Surgical treatment

**Fig. 4:**
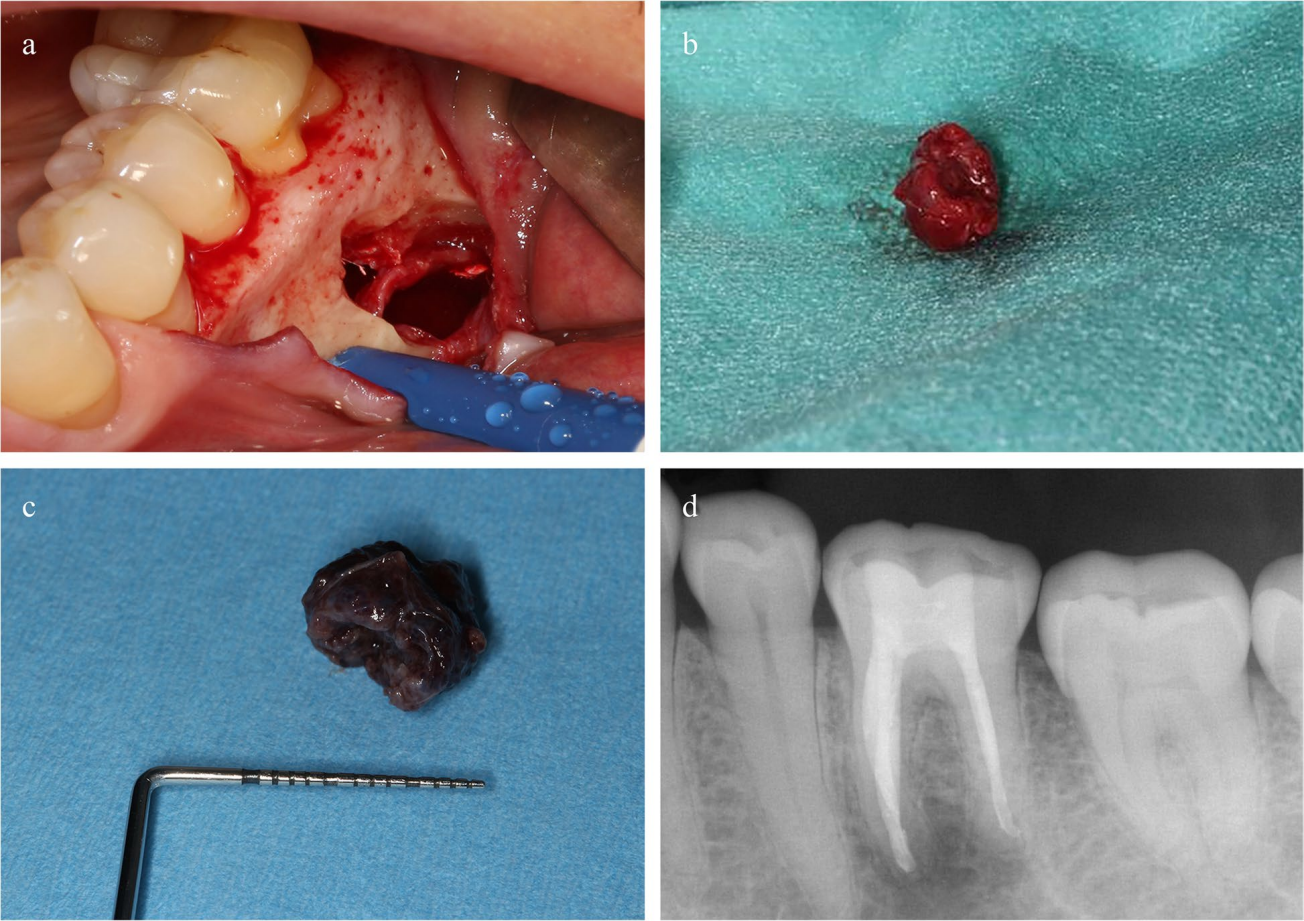
Intra/ extra-oral color photograph and periapical radiographs (Canon EOS 70D and Canon Macro Ring Lite MR-14EX, Canon, Tokyo, Japan). **a** Removed hemangioma. **b** Hemangioma after being fixed in formalin with the size of nearly 12 mm. **c** Clean intrabony cave depicting centrally the inferior alveolar nerve. **d** IOPAR after apicoectomy

One week after termination of the root canal treatment, surgery was scheduled in order to finally clarify the diagnosis of this osteolytic process. Due to a respiratory infection of the patient, surgery was postponed for another week. Because of the findings collected during clinical and radiographic examinations as well as endodontic treatment, the surgical procedure was planned to be carried out as minimally-invasive and tissue conservative as possible. At that time, the most probable diagnosis was that of an infected radicular cyst proceeding from the mesial root of tooth 36. This diagnosis was possible despite the prior positive pulp sensibility of teeth 36 considering that only the mesial root was affected by the lesion and the vitality of the distal root was responsible for the results of the pulp sensibility test. Prior to the start of the surgical procedure, again the sensibility of the mental nerve was tested as well as the sensibility of the teeth 34, 35, and 37. No limitations could be detected. Local anaesthesia was performed with articaine with supplement of epinephrine 1:100,000 (Ultracain forte, Sanofi-Aventis, Frankfurt, Germany) via infiltration anaesthesia. The design of the full thickness flap was as minimally-invasive as possible. The papilla 34 to 35 was detached, marginal incision was made along 35 to 36. Due to the close anterior positional relationship of the mental foramen to the space-consuming lesion, the decision was reached to perform a distal vertical relief incision, which was directly distal from the papilla 36 to 37. The mucoperiosteal flap was elevated with a raspatory, followed by blunt preparation with a gauze pad up to 1 cm near the expected mental foramen. Thereby, the nerve was gently exposed and could be protected by the use of a narrow, blunt retractor. The bony access was chosen in a more distal position above the suspected space-occupying lesion, and initially prepared with a rose head bur followed by a diamond bur after the color of the bone changed to darker. When the covering bone, which was consecutively put into 4 % formalin and turned in for histopathological examination, was completely removed, the dark red color of the lesion appeared. Surprisingly, the consistence of the lesion seemed not to be firm elastic as expected for an anticipated cystic lesion. In the contrary, the lesion appeared very solid, it was hardly possible to achieve a slight impression. At this point, it was clear that the originally planned surgical intervention could not be an ordinary biopsy. Moreover, due to the consistence and topographic location of the tissue in the lesion, it appeared also obligate to avoid an incision biopsy to minimize any risk for the alveolar nerve. The risk of an axonal injury was too high, because of the obscure position of the mental nerve. Therefore, the original plan to take a surgical probe excision, was neglected and in lieu thereof the entire removal of the tissue was intended. Thus, different sinus-lifting elevators (Frios^â^SinusSet, Dentsply IH GmbH, Mannheim, Germany) were used to carefully prepare the lesion in the posterior region. It was possible to detach the mass off the bony wall. In combination with gentle swinging and shaking movements the inferior alveolar nerve could be depicted in the posterior region. Integrity of the nerve could be controlled via mild pulling on the nerve covering tissue and observing the little movements outside the mental foramen. The same procedure was performed in the anterior part of the bony cavity until the nerve was depicted here also. The strong adherence of the nerve enclosing tissue was detached by spreading the mass with tweezers and blunt preparation with a scissor precisely to the border of the nerve sheath, beginning from the edges to the centre. Finally, both structures could be separated, whereupon the nerve was exposed in the middle of the bony cavity without any conspicuous damages or alterations of the nerve sheath (Fig. 4a). The removed tissue was also put into 4 % formalin and turned in for histopathological verification (Fig. 4b, c). A minimally-invasive apicoectomy of the mesial root of tooth 36 was performed. The distal root tip remained untouched during surgery and was purely treated with an orthograde root filling prior to the surgical intervention. As an X-ray prior to the surgical intervention showed that the sealer of the mesial root has been overfilled apically in terms of the orthograde filling though (Fig. 3d), the exceeding sealer was cured with access from the lesion’s lumen intraoperatively and the root tip was then minimally reduced by using surgical drills. Since the orthograde root canal fillings were of high quality and very solid, no retrograde cavity with following filling were implemented (Fig. 4d). The bony wall of the defect was gently scraped with a sharp spoon without any contact to the nerve. Thereby, a good blood seepage was achieved. The access flap was adapted with single button sutures (Ethibond 5-0-, Ethicon/ Johnson & Johnson, Neuss, Germany) in a more coronal position to cover the former vestibular gingival recession the teeth 45 and 46. Subsequently, after surgery, pain stimulus caused by stitches with a dental probe was realized by the patient.

## Histopathological findings

**Fig. 5:**
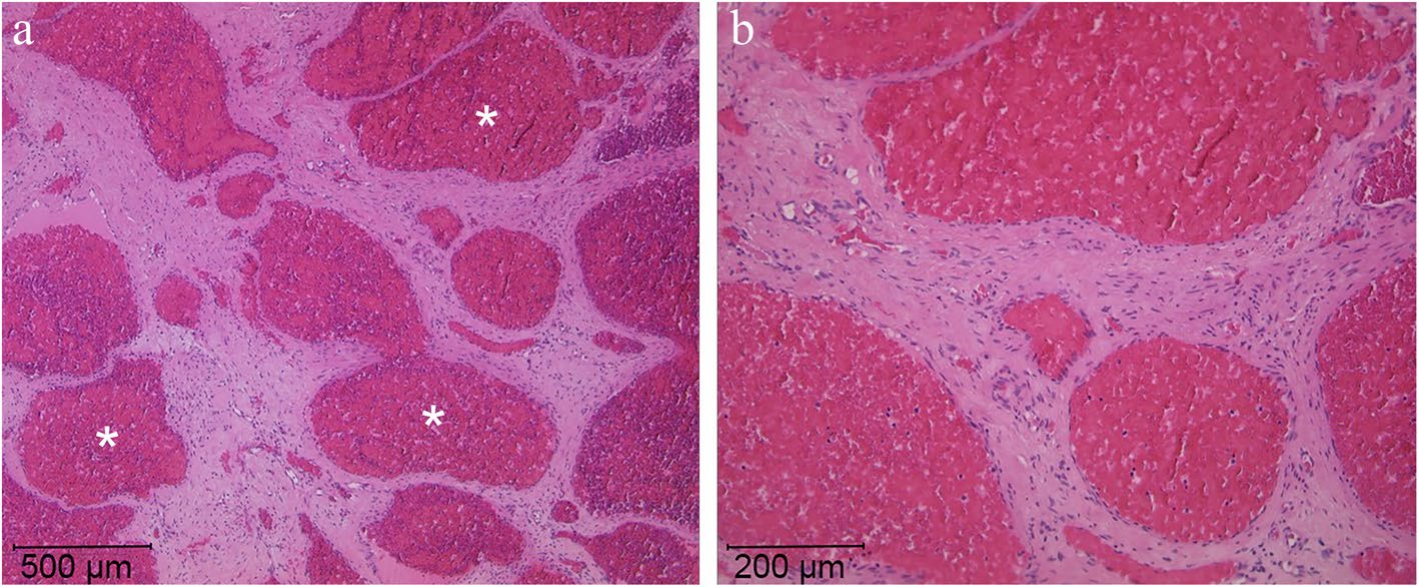
Histomorphology of intraosseous haemangioma. **a** Large cystically dilated blood vessels (star) filled with erythrocytes and surrounded by a fibrous stroma (HE, 50x). **b** On higher magnification, the dilated blood vessels are lined by a thin wall of non-atypical endothelial cells with small nuclei (HE, 100x)

The intraoperative tissue samples of the space-occupying lesion were processed and evaluated at the Department of Pathology. The tissue exhibited large, irregular, dilated blood vessels filled with erythrocytes, and surrounded by a fibrous stroma (Fig. 5a). At higher magnification, the dilated blood vessels were lined by a thin wall of non-atypical endothelial cells (Fig. 5b). Based upon the histomorphological evaluations, the lesion was diagnosed as hemangioma. No signs of malignancy were found.

### Follow-up controls

As early as in the evening after surgery the patient reported that sensibility in the lower lip was subjectively recovered. Wound healing period was completely uneventful and without any discomfort. Twelve days after surgical treatment, no signs of inflammation such as swelling could be detected so sutures were removed. Recall intervals were scheduled at every six weeks for the first seven months. Sensibility testing of teeth 33, 34 as well as 35 were positive and provocation via percussion on the teeth in the left mandible was unobtrusive within the whole recall period.

**Fig. 6:**
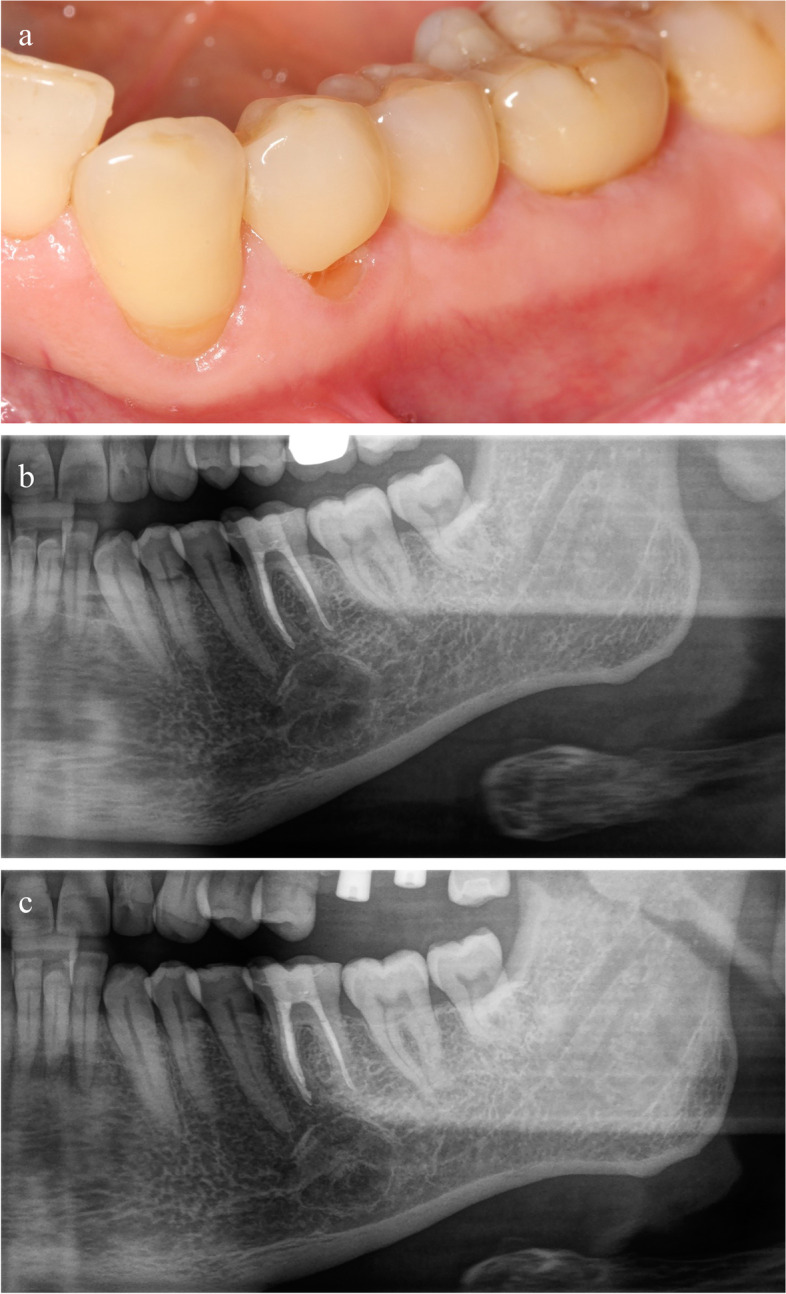
Intra-oral color photograph and partial panoramic radiographs follow-ups. **a** Clinical audit 7 months post-surgery. **b** Partial panoramic x-ray 7 month postoperative. **c** Partial panoramic x-ray forty-five months postoperative

Seven months after surgical procedure, an intact bone contour in the region of the former bony window was clinically palpable. Up to that point, the patient reported a slight numbness in the left mental dermal region, but no pain at all. The objective verification did not show any sort of numbness. Both, discrimination between sharp and blunt object as well as two-point discrimination were reproducible at 100%. In addition, slightest contacts in the left mental dermal region were realized by the patient without any difference to the corresponding right areas. The operation field itself was completely inconspicuous and the mucosa revealed no scars (Fig. 6a). A partial panoramic radiograph of the left side seven months postoperative illustrates a reorganisation of the bony defect (Fig. 6b).

The twelve months post-excision control demonstrated a further improvement with respect to the subjective dermal sensibility in the mental region, whereas in the patient’s opinion it was not yet equal to the contralateral side. Similar to the seven-month control, the patient precisely realized the objective verification measured with stump vs. sharp testing. The further panoramic radiograph twelve months postoperative presents a similar impression of the region of interest as the post- seven-months radiograph. A remarkable inhomogeneous reorganisation of the bony defect is observable including an increase of radio-opacity. Forty-five months after surgery, a panoramic radiograph of the left mandible was performed. No signs of recurrence could be observed. The former lesion showed increased radiopacity while the border of the lesion decreased and got more diffuse (Fig. 6c).

Moreover, the clinical situation appeared unobtrusive. Stable and unaltered sharp and blunt discrimination as well as two-point discrimination were reproducible in 100%. Slightest touches were also perceived. No signs of swelling or discoloration could be determined. Table 1 depicts the timeline of the present case.

**Table 1:** Timeline of the present case

Date	Appointment	Procedure
11/2017	First routine dental examination	Clinical examination, dental pulp sensibility testing, dermal sensibility testing, panoramic radiograph, x-rays of tooth 36
03/2018	Root canal treatment	Clinical examination, dental pulp sensibility testing, dermal sensibility testing, x-rays of tooth 36
04/2018	Surgical intervention with apicoectomy of the mesial root	Clinical examination, dental pulp sensibility testing, dermal sensibility testing, panoramic radiograph, x-rays of tooth 36
04/2018	12-days post-excision control	Clinical examination, dental pulp sensibility testing, dermal sensibility testing
06/2018	2-months post-excision control	Clinical examination, dental pulp sensibility testing, dermal sensibility testing
08/2018	4-months post-excision control	Clinical examination, dental pulp sensibility testing, dermal sensibility testing
11/2018	7-months post-excision control	Clinical examination, dental pulp sensibility testing, dermal sensibility testing, partial panoramic radiograph left mandible
04/2019	12-months post-excision control	Clinical examination, dental pulp sensibility testing, dermal sensibility testing, panoramic radiograph
01/2022	45-months post-excision control	Clinical examination, dental pulp sensibility testing, dermal sensibility testing, panoramic radiograph of the left mandible

### Patient perspective

Keeping in mind the last forty five months, the patient summarizes the entire interdisciplinary endodontic and surgical intervention including the follow-up examinations to be successful. Apart from the complication-free and scarless wound healing, the patient subjectively reports of a discrete irritation during shaving within the left mental region.

## Discussion

The present case illustrates a rare example of an intraosseous hemangioma, which has been well-documented with respect to its interdisciplinary treatment and its follow-up history. In the current case, the diagnostic finding was an incidental finding after fabrication of a panoramic x-ray. In 2007, the patient firstly attained knowledge of an intrabony osteolytic process in the left mandible. At that time point as well as ten years later, both panoramic radiographs showed a homogeneous, unilocular, well-defined osteolytic lesion. This is in contrast to the radiological appearance of most of the intraosseous hemangiomas, which is described as soap bubble, honeycomb-like appearance, a sunray appearance, caused by bony trabeculae in the lesion or a poorly defined osteolytic lesion [14, 15]. The variable appearance of central hemangioma in different projections was also described [16]. Differential diagnosis of for example aneurysmal bone cyst, fibrous dysplasia, ameloblastoma, osteoma, osteosarcoma, giant cell lesion, residual cyst, multiple myeloma, and myxoma could not be radiographically distinguished [17]. Therefore, it is not possible to diagnose an intraosseous hemangioma based on dental radiology alone. Clinical findings are also varying. Many patients do not report about any symptoms, as described in the current case. But bony swellings of different size, sometimes causing asymmetry of the face, paraesthesia of lips as well as the mental region, pulsation or discomfort can occur as well [12, 18–20]. Symptoms regarding teeth and the alveolar process such as bluish discoloration of the gingiva, mobile or displaced teeth, agenesis of teeth, alteration of the dental arch or seeping gingival bleeding which were also described, were not found in the present case. Moreover, the patient did not report about early eruption of the permanent teeth, which could also be related to central hemangiomas of the jaw [11, 18, 21–24]. Osteolytic lesions that are of extraordinary or inexplicable origin, as depicted in the current case, should be biopsied and clarified [24]. From a critical point of view, advantages of intraoperative biopsies are to be contrasted with the disadvantages of the same. The main advantage of a biopsy is seen in the histological verification of a diagnosis. The so collected details allow a precise surgical procedure according to the respective pathology. As a possible disadvantage of a biopsy, severe complications could result from the lack of knowledge regarding the lesion. For instance, if an intraosseous hemangioma is present like in the present case and a biopsy would have been taken, serious complications in form of severe bleeding would be possible during surgery or diagnostic confirmation. It was described that patients died after extractions of teeth with hemangioma association or even after puncture biopsy of a hemangioma of the jaw [10]. For this reason, it is important to be as careful as possible during surgery and to gently remove every tissue layer, without touching the lesion itself, as it was performed in the current situation. The vestibular access provided an optimal overview and allowed to depict the mental foramen for estimation of the mandibular nerve pathway. This was also necessary to protect the mental nerve. If hemangiomas, especially extensive ones, are expected because of radiological appearance and clinical signs, angiography before surgery could be a very useful tool for detecting feeding vessels and planning the approach of therapy [25]. In the present case, it was assumed that the lesion would be a sort of cyst. Accordingly, there was no need to apply angiography. Treatment options that provide complete histopathological investigation reach from partial mandibuloectomy to minimally invasive approaches such as curettage or surgical removal of the lesion with or without ligation or embolization of the feeding vessels [11]. The option, to omit any invasive intervention, in particular, in the present case could have led to root resorptions of neighboring teeth [26], a compression of the inferior alveolar nerve accompanied by paresthesia or pain, and a further expansion to the mental foramen, where a removal would be much more complicated or a reduction of the lower jaw stability [27]. However, since the patient was originally asymptomatic for more than ten years, it was an option to left the lesion untreated [28]. Indeed, the consequence, to clarify the diagnosis of the lesion, relied on the fact, that the inferior alveolar nerve was not detectable within the CBCT. The plan of sample taking in order to histologically verify the most probable diagnosis of an infected radicular cyst, was rejected intraoperatively based on the atypical appearance of the lesion. Instead, the decision was made to completely remove the lesion, carefully as recommended by other authors [11, 18].In the present case, the size of the mass as well as its texture allowed for a gentle procedure resulting in a preservation of teeth and the mental nerve resulting in a nearly four years follow-up without recurrence of the intraosseous lesion.

## Conclusion

Dentists, oral surgeons and maxillofacial surgeons have to be aware of clinical symptoms and radiological appearance of intraosseous hemangiomas to avoid severe complications even though these lesions occur very seldom in the jaws. Gentle and minimally invasive surgery protects neighbouring structures like teeth or nerves completely. Moreover, for the conservative dentist, this case report impressively supports the fabrication of a panoramic x-ray in addition to bite wings being made for the assessment of carious lesions during the first appointment of a new patient. As seen in the present case report, without doing so, the hemangioma would have been overlooked, perhaps resulting in serious consequences for the patient.

## Data Availability

Not applicable.
